# Laboratory Validation of Instrumented Mouthguard for Use in Sport

**DOI:** 10.3390/s21186028

**Published:** 2021-09-09

**Authors:** Danyon Stitt, Nick Draper, Keith Alexander, Natalia Kabaliuk

**Affiliations:** 1 Department of Mechanical Engineering, University of Canterbury, Christchurch 8041, New Zealand; danyon.stitt@pg.canterbury.ac.nz (D.S.); keith.alexander@canterbury.ac.nz (K.A.); natalia.kabaliuk@canterbury.ac.nz (N.K.); 2 School of Health Sciences, University of Canterbury Christchurch, Christchurch 8041, New Zealand

**Keywords:** instrumented mouthguard, head impacts, concussion, accelerations, sports

## Abstract

Concussion is an inherent risk of participating in contact, combat, or collision sports, within which head impacts are numerous. Kinematic parameters such as peak linear and rotational acceleration represent primary measures of concussive head impacts. The ability to accurately measure and categorise such impact parameters in real time is important in health and sports performance contexts. The purpose of this study was to assess the accuracy of the latest HitIQ Nexus A9 instrumented mouthguard (HitIQ Pty. Ltd. Melbourne Australia) against reference sensors in an aluminium headform. The headform underwent drop testing at various impact intensities across the NOCSAE-defined impact locations, comparing the peak linear and rotational acceleration (PLA and PRA) as well as the shapes of the acceleration time-series traces for each impact. Mouthguard PLA and PRA measurements strongly correlated with (R^2^ = 0.996 and 0.994 respectively), and strongly agreed with (LCCC = 0.997) the reference sensors. The root mean square error between the measurement devices was 1 ± 0.6g for linear acceleration and 47.4 ± 35 rad/s^2^ for rotational acceleration. A Bland–Altman analysis found a systematic bias of 1% for PRA, with no significant bias for PLA. The instrumented mouthguard displayed high accuracy when measuring head impact kinematics in a laboratory setting.

## 1. Introduction

Concussion is an inherent risk of participating in contact, combat, or collision sports. All attract high numbers of participants, with over 23 million registered participants internationally [1,2,3,4,5]. Given the high intensity of these sports, injuries are common, with concussions being one of the most common. One study estimated as many as 300,000 sports-related concussions occur annually in the US alone [6], creating an associated economic burden of USD 60 billion annually [7]. Numerous researchers have investigated concussion rates, and reports vary, with 0.5–250.6/1000 athlete exposures (AE) [8,9,10,11,12,13,14,15,16,17]. An AE is defined as one person participating in one game or match. For example, one boxing match has two AEs.

When the head experiences an impact, the varied density causes parts of the brain to accelerate at different rates, deforming the tissue [18]. The brain can handle some deformation; however, once a certain threshold is surpassed, trauma occurs, eliciting a variety of biological responses [18]. Impacts over 10 *g* not resulting in acute concussion symptoms have been labelled subconcussive impacts [19]. It has been suggested that combinations of concussive and subconcussive head impacts may result in long-term conditions such as chronic traumatic encephalopathy [20], cognitive impairment [21], and depression [22]. Most concussions go unreported until symptoms show, which can be up to a few days [23], meaning a large number are not identified until 24 hours or more after the injury [24,25]. Additionally, reports suggest that approximately 90% of concussions do not result in a loss of consciousness [23,26], further complicating on-field diagnosis. As a result, underreporting rates are estimated to be as high as 50–90% [26]. For 80 to 90% of cases, the individual’s symptoms resolve within 2 weeks [25,27,28,29,30], although recovery appears to be slower for adolescents than college-age athletes [28,31,32,33].

In an ideal case, assessment of injured players is facilitated by a certified athletic trainer, team physician, or health care provider on the sideline; however, the vast majority of young athletes practice and play in circumstances in which trained personnel are not immediately available [18]. In New Zealand, sporting guidelines state that players suspected to have sustained a concussive injury are to be removed from play immediately and monitored for symptoms [23]. This method relies upon the player correctly reporting the immediate symptoms, which hinders effectiveness, as there is a tendency for athletes to underreport their symptoms [34,35,36].

Wearable sensor technology exists that can report real-time impact information. The most common types are helmet-integrated sensors, patch sensors, and mouthguard sensors, all of which measure peak linear acceleration (PLA), rotational velocity (PRV), and/or rotational acceleration (PRA). The first is the head-impact telemetry (HIT) system, finding common use in studies of American football or ice hockey. Using a 12-accelerometer array embedded rigidly in hard-shelled helmets, accuracy of the device depends on the fit of the helmet [37,38]. Patch and mouthguard sensors can be used in unhelmeted sports such as football or rugby. Patch sensors are secured on the skin using an adhesive or a headband, and are commonly placed over the mastoid process behind the ear [37]. They measure head impacts using three linear accelerometers and a triaxial angular rate sensor. Laboratory validation studies have found the patch sensors to perform poorly. One study found the X-patch to have an error rate of up to 50% for PLA and PRA [39], with another finding the patch to underestimate PRA by more than 25% on average [40]. A 2017 study found the patch to have root mean square (RMS) errors of 34% for PLA and 23.4% for PRA [41].

In vivo validation studies have reported similar results. Both patch and headband sensors showed large errors due to slipping over the skull with the skin, giving false acceleration values [42]. This study reported movements during impacts of 2−4 mm for the patch sensors and 2–13 mm for the headband sensor, while the mouthguard sensor showed far less movement (<1 mm) relative to a marker secured in the ear [42]. Additionally, video validation found the X-patch had a positive predictive value of 16.3% for head impacts [42]. Comparatively, a laboratory validation study of the X2 mouthguard reported average RMS errors of 9.9 ± 4.4% for linear acceleration (LA) and 9.7 ± 7% for rotational acceleration (RA) [43]. However, the X2 performed poorly when estimating impact directions [44].

A new instrumented mouthguard, the HitIQ Nexus A9, has recently been developed by HitIQ Pty Ltd. The Nexus A9 boasts two independent measurements of rotational kinematics, which may allow the mouthguard to display increased accuracy and dynamic range of angular kinematic estimates. However, being relatively new to the market, this mouthguard currently lacks validation in a laboratory setting. Instrumented mouthguards have been shown to produce lower errors and have better coupling to the head compared to other systems, thus proving to be the best method for identifying concussive impacts during gameplay. The purpose of this study is therefore to assess the accuracy of the HitIQ Nexus A9 instrumented mouthguard in a controlled laboratory setting. This will be achieved by comparing the PLA and PRA values and shapes of the acceleration time-series traces of the mouthguard sensor to a reference accelerometer pack inside a 50th-percentile ATD headform.

## 2. Materials and Methods

### 2.1. Experimental Procedure

**Figure 1:**
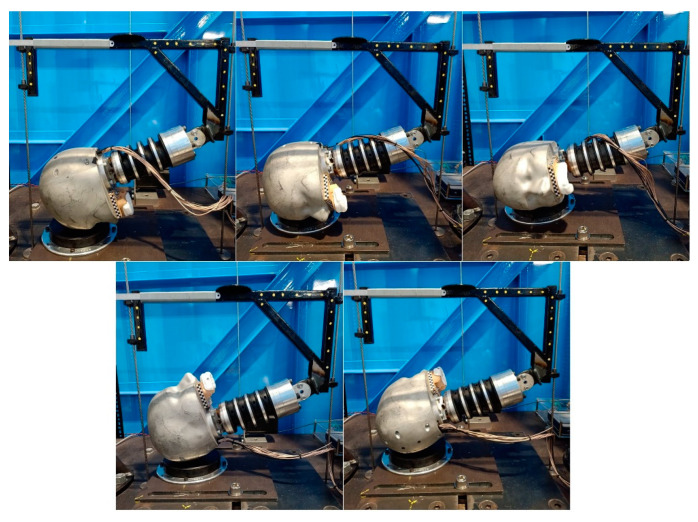
Impact locations, from top left to bottom right: front/forehead, front boss, side, rear, and rear boss. The mouthguard and dentition are both shown rigidly fixed to the upper jaw of the ATD headform.

Testing was carried out using a gravity-induced, twin-wire-guided drop-test rig. An electromagnet lifted and held the drop carriage shown in Figure 1, then released it from the specified height. The impact locations were: front, front boss, side, rear, and rear boss, as described by the NOCSAE standard [45] (Figure 1). The headform used was based on the Hybrid III 50th-percentile male headform (Humanetics group, Farmington Hills, MI, USA.), modified to include a rigidly fastened dentition (Figure 1). This headform was made of aluminium and excluded the rubber skin cover found on the Hybrid III headform. The neckform used was a Hybrid III neckform replica.

**Figure 2:**
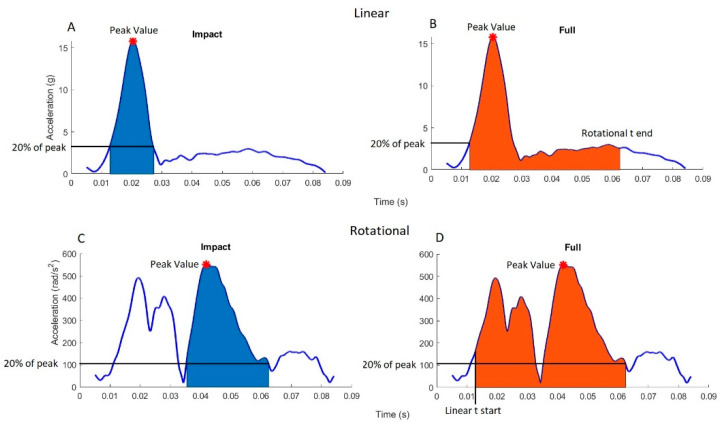
Time periods considered for the RMS calculations, with Impact (**A**,**C**) defining a threshold of 20% of the peak on either side of the peak, and Full (**B**,**D**) defining the same time duration for both based on linear and rotational 20% thresholds.

Drop heights were established to achieve PLAs of 20, 40, and 80 g; these were chosen because they fell within the range of published data for sporting head impacts [10,46,47,48,49,50]. Three impact durations for the LA time-series traces were targeted. These were 15, 30, and 60 ms, to represent short-, medium-, and long-duration head-acceleration events, respectively (Table 1). Test durations were defined based on a threshold LA of 20% of the PLA on either side of the peak (Figure 2a). Certain high-energy impact scenarios were not performed due to the likelihood of damaging components.

The headform was dropped onto a 1 inch modular elastomer programmer pad (Cadex Inc., Quebec, QC, Canada), which was chosen because it displays a similar impact response across a wide range of strain rates, and is commonly used throughout the literature. On top of this pad, various combinations of foam were placed to achieve the desired PLAs and durations. Foam combinations were coded A–L for ease of display; detailed lists can be found in Appendix A and Appendix B. Drop heights were taken from the top of the impact surface to the lowest point of the headform when attached to the drop carriage. Each test impact scenario was repeated five times.

### 2.2. Mouthguard

The HitIQ Nexus A9 instrumented mouthguard (HitIQ Pty. Ltd., Melbourne Australia) is relatively new to the market, and as such has not previously undergone validation in a laboratory setting. The mouthguard included an array of three accelerometers (Analog Devices (Norwood, MA, USA) ADXL372, range: ±200 g, 12-bit) and a gyroscope (Bosch (Gerlingen, Germany) BMG260, ±2000 dps range, 16-bit), providing two independent measurements of rotational acceleration and velocity, respectively, which may allow the mouthguard to display increased accuracy and dynamic range of the angular kinematic estimates. A three-accelerometer array also provided an estimate of angular acceleration independent of the gyroscope, allowing for a cross-check to remove spurious readings originating from actions such as mouthguard deformation, rather than head kinematics. Two accelerometers were located on the outside of the first molar, on both the left and right sides of the mouthguard. The third accelerometer and a gyro were mounted on the inside of the central incisors.

The mouthguard componentry was mounted to a base layer of high-shore-rated ethylene vinyl acetate copolymer (EVA) mouthguard material that was thermoformed to a 3D-printed high-resolution dentition. It was secured to the base layer by thermoforming a hard and thin intermediate layer of styrene–butadien–styrene (SBS), which protects componentry and the users’ teeth. A thicker final layer of EVA was then thermoformed over this, which reinforced the support of the intermediate layer and componentry. The bonding of the intermediate layer around the componentry, along with EVA hot melt used around componentry edges, ensured the componentry would remain in position. The base layer and final layer had enough surface area around the componentry to bond to each other under heat applied during manufacturing, ensuring that the componentry was fully encapsulated and tightly sealed within the mouthguard material. The total thickness of these layers was up to 5.8 mm. All materials complied with the biocompatibility standard ISO10993, and were approved dental/medical grade materials.

The sensors were each located in a different orientation. As a consequence, each local reference system was rotated with respect to the reference system at the centre of the head. Calibration of the sensors was carried out using a specialized apparatus and software to compare sensors to a known reference. The gains and transformations needed to rotate each sensor’s signals from the local sensor system of reference to the system of reference at the centre of the head could then be found. Subsequent to this, kinematic equations (3D) of the head were calculated to ensure that all sensors projected the same linear acceleration to the centre of the head.

### 2.3. Data Acquisition

The headform housed four tri-axial accelerometers (Analog Devices (Norwood, MA, USA) ADXL377, range: ±200 G, sensitivity: 6.5 mV/g) for a total of 12 sensing axes. Accelerometers were configured in a standard "nine-accelerometer package" (NAP) array [51], with the three redundant sensing axes configured radially along each primary axis. The data were recorded using a NI9205 analog input module (National Instruments (Texas, TX, USA), sample rate: 20 kHz, 16-bit) and stored via a LabVIEW program. Accelerometer data were processed to determine the linear and rotational acceleration at the centre of mass of the headform, according to the standard NAP algorithm [51]. Once a kinematic solution was found, results were projected back to the location of each accelerometer and cross-checked with their actual reading, thereby allowing identification of capture errors such as misalignment and deformation.

The gyroscope and accelerometers were sampled at 800 Hz and 3200 Hz, respectively, to reflect the different spectral components of on-field impacts determined experimentally through field trials in Australian Rules, Rugby League, and American Football. A dentition, to which the mouthguard was moulded, was attached to the upper-jaw region of the headform (Figure 1). The dentition was bolted to the headform, and tightness of the bolts was checked periodically. The mouthguard had an indicative trigger level set at 10 G, and was set to record 20 ms of data before the first trigger and 80 ms after the last trigger in the event. A retrigger function allowed the capture of impact events containing complex kinematics, compared to a fixed-length window that can lose context during complex, multi-impact events. Data recorded by the mouthguard was saved temporarily to onboard flash memory. Then, that data was uploaded to the same computer that processed the NAP data. This data was referenced with the impact parameters (PLAs, impact duration, impact location, etc.).

### 2.4. Post-Processing

Time-series trace data for both linear and rotational acceleration was collected in MATLAB, and then was used to find the resultant linear and rotational accelerations. For each separate impact, PLA and PRA (Figure 2) were defined as the maximum values of the resultant time-series data for linear and rotational acceleration, respectively. These were used for the regression and Bland–Altman (BA) analyses. Similar to previous studies [43,52,53], the RMS error was calculated between the reference and mouthguard traces for both linear and rotational acceleration. Signals were first temporally aligned such that minimal area existed between them, following which the RMS and normalised RMS (NRMS) errors were calculated using Equations (1) and (2):

(1) $$RMS=\sqrt{\frac{\sum_{i=1}^{n}{\left(x_{i}^{mg}-x_{i}^{ref}\right)}^{2}}{n}}$$

(2) $$NRMS=\frac{RMS}{max_{ref}}\times 100$$

where *n* is the number of measurements; $x_{i}^{mg}$ and $x_{i}^{ref}$ are the measurements made by the mouthguard and reference sensors, respectively; and $max_{ref}$ is the peak reference acceleration. Two methods of trimming data for the RMS calculations were used. The first trimmed the data at a threshold acceleration of 20% of the peak on either side of the peak of the reference time-series trace, called ‘Impact’ RMS (Figure 2A,C). The second trimmed the data to start at a threshold of 20% PLA before the linear peak, and end at a threshold of 20% PRA after the rotational peak, called ‘Full’ RMS (Figure 2B,D). A 20% threshold was experimentally determined to include the necessary trajectories generated by the impact rig, whilst excluding largely noisy data pre- and post-impact. The ‘Impact’ period was expected to be the impact part associated with the highest risk of brain injury, and has been commonly used in previous studies; however, fidelity of the ‘Full’ model may be required when investigating subconcussive impacts, in which impact energy is spread over a longer time.

### 2.5. Statistical Analysis

The statistical analysis was carried out in MATLAB (R2019) with an α *p* ≤ 0.05 for accepting statistical significance. Peak value correlation was assessed using linear regression, and coefficients of determination (R^2^) for linear and rotational acceleration. Bland–Altman analyses were used to assess levels of agreement (LOAs, 95%) and assess any systematic bias of measured PLA and PRA between the reference and mouthguard [54,55]. Lin’s concordance correlation coefficients (LCCC) and associated 95% confidence intervals were also calculated to assess agreement between the two sensor systems. Paired-sample t-tests were calculated for RMS error values to assess statistically significant differences (*p* ≤ 0.05) between the ‘Impact’ and ‘Full’ RMS and NRMS. The accompanying *p*-values and effect size using Cohen’s d values were also calculated. A Cohen’s d value of ≤0.2 implies a weak effect, 0.5 implies a medium effect, and ≥0.8 implies a large effect. Strength of agreement criteria for LCCC was put forward with substantial agreement at LCCC = 0.95–0.99, and ‘almost perfect’ agreement at LCCC > 0.99 when compared to the lower one-sided 95% CI [56].

## 3. Results

**Figure 3:**
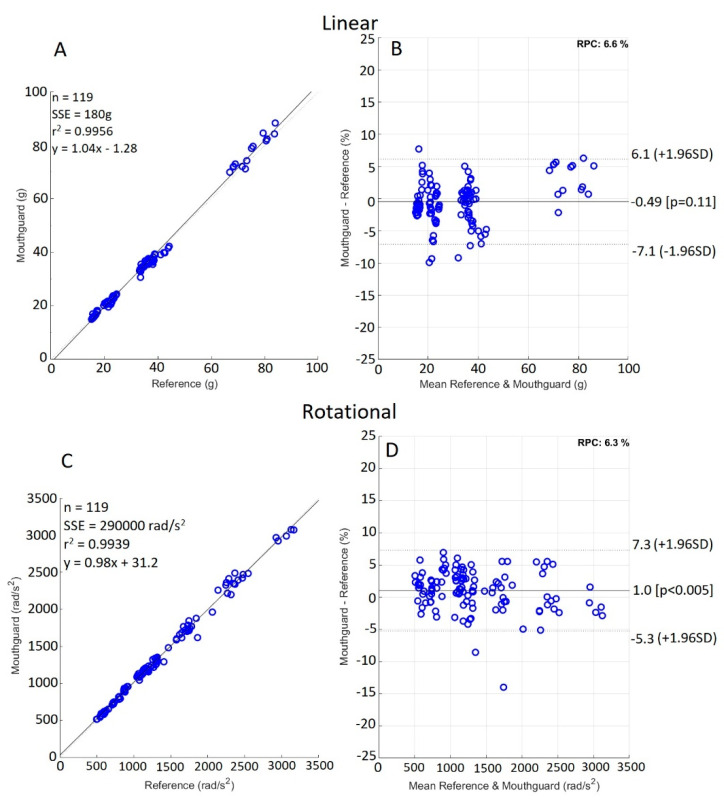
Correlation (**A**,**C**) and Bland–Altman (**B**,**D**) plots for PLA and PRA.

Differences in the peak value error between impact locations were small (mean (SD): 0.66% (2.28%) for relative PLA, and −1.21% (−0.86%) for the difference in relative PRA error between impact locations), therefore results were pooled together. Figure 3a–d display the correlation and BA scatter plots for PLA and PRA. The peak values measured by the mouthguard showed strong positive correlation with those of the reference sensors (R^2^ = 0.996 for PLA and 0.994 for PRA). Figure 3b shows a nonsignificant bias of −0.49% (*p* = 0.11) for PLA, and Figure 3d shows a significant bias of 1% for PRA (*p* < 0.05). Limits of agreement were similar between PLA and PRA (+6.1%, −7.1% for PLA; and +7.3%, −5.3% for PRA). Figure 3d shows one outlier, representing a rear boss, 40 g, 15 ms impact with a 13.1% mouthguard PRA underestimation.

**Figure 4:**
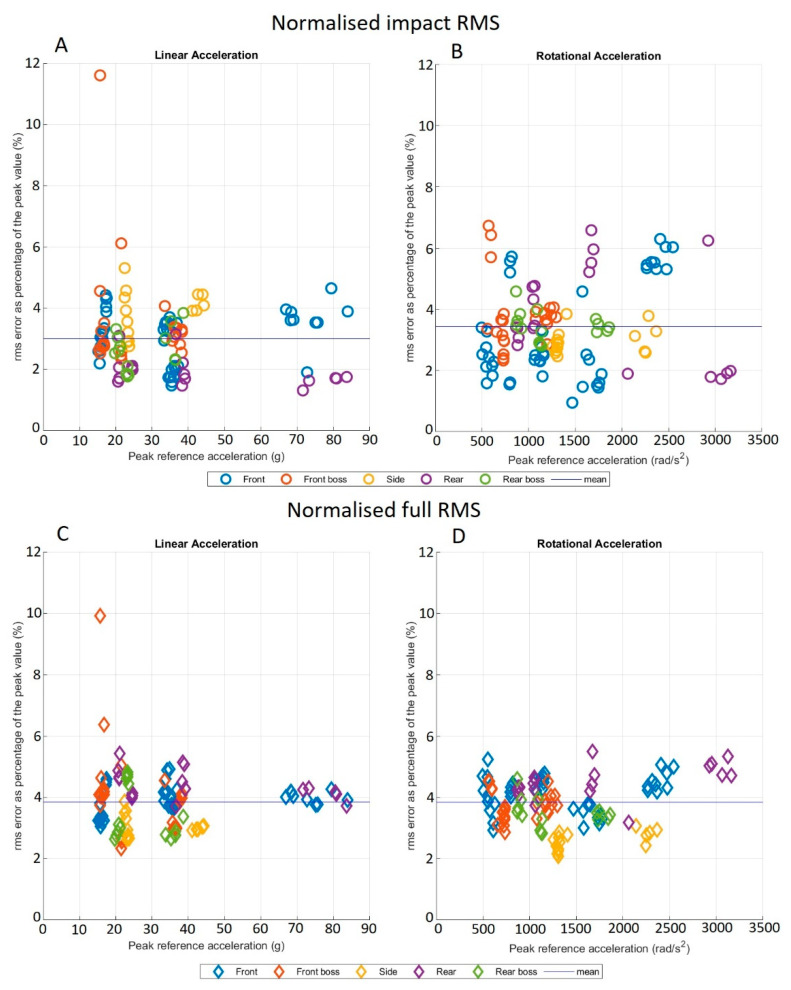
Normalised RMS values calculated over two different time periods for both linear and rotational accelerations. (**A**,**B**) Normalised impact RMS, (**C**,**D**) Normalised full RMS. The blue line shows the respective means. The colors show the impact location for each of the impacts.

**Figure 5:**
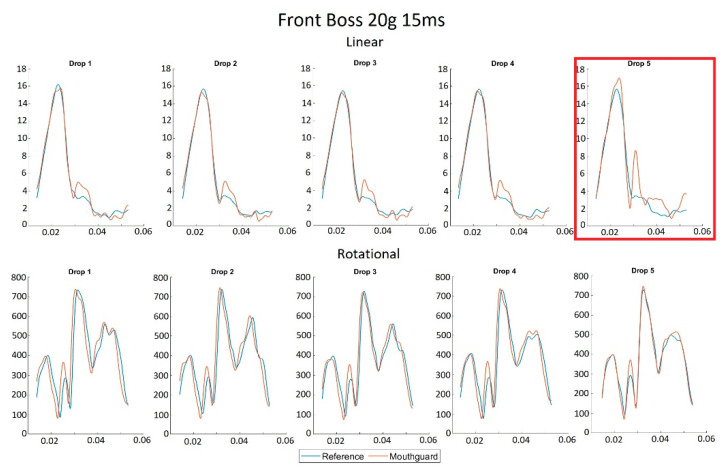
Each impact in the 20 g, 15 ms front boss scenario with the outlier marked.

Figure 4A–D show the ‘Impact’ and ‘Full’ NRMS errors across each impact location. In both cases for LA, one outlier was seen. The associated time-series traces are shown in Figure 5. The two time periods over which the RMS and NRMS were calculated produced different results, with the ‘Full’ RMS producing consistently higher RMS values for both linear and rotational acceleration. Paired-sample t-tests revealed mean differences between the two periods of 0.31 g for LA RMS (t_(109_ = 5.52, *p* < 0.05, d = 0.54), –5.50 rad/s^2^ for RA RMS (t_(109)_ = 1.65, *p* = 0.10, d = 0.18), -0.85% for LA NRMS (t_(109)_ = 5.88, *p* < 0.05, d = 0.56), and 0.41% for RA NRMS (t_(109_) = 3.01, *p* < 0.05, d = 0.3). Figure 4 shows that there was no visible increase in the NRMS as the PLA increased. Additionally, the NRMS showed no relationship with impact duration.

Figure 5 shows the NRMS outlier, a 20 g, 15 ms front boss impact. This was the only outlying impact of the five recorded for that scenario. The high NRMS error was caused by the recognition of a second apparent impact after the initial peak, likely due to a minor decoupling of the mouthguard from the dentition or a slippage of the foam impact surface. This was only displayed in the linear acceleration time-series trace for this impact.

Minimal differences were found between the correlation coefficients of linear and rotational accelerations (Table 2). There was no significant difference (t_(__118)_ = 0.42, *p* = 0.68, d = 0.11) between the relative peak acceleration errors for linear and rotational accelerations. Table 2 also shows the means and standard deviations of peak and RMS errors, found by taking the mean and standard deviation of all the impacts.

## 4. Discussion

The results showed a strong linear correlation between the Nexus A9 mouthguard and the headform reference sensors for both PLA and PRA, with R^2^ values of 0.996 and 0.994, respectively. The mouthguard showed strong agreement with the reference sensors, with an LCCC value of 0.997. A study conducted by Greybe et al. utilised a pendulum impactor, producing PLAs of 7–102.5 g, when comparing an intelligent mouthguard to reference sensors inside a headform [52]. The R^2^ values were 0.93 for PLA and 0.99 for peak rotational velocity (PRV), whilst PRA was not investigated. An earlier mouthguard validation study by Bartsch et al. utilised both a headform simulation and in vivo head impacts, producing R^2^ values of 0.99 for PLA, 0.99 for PRV, and 0.98 for PRA [53]. A third mouthguard validation study by Camarillo et al. reported R^2^ values of 0.96 for PLA, 0.89 for PRA, and 0.98 for PRV [43]. Typically, the strongest correlation for sporting head-impact sensors are PRV values, with most studies reporting R^2^ values of 0.98–0.99 [40,41,43,57], whilst PRA tends to have a weaker correlation with reference sensors.

The BA analysis showed a nonsignificant (*p* = 0.11) bias of −0.49% with 95% LOA of (−7.1, 6.1%) for PLA, and a statistically significant bias of 1% with 95% LOA of (−5.3, 7.3%) for PRA. The PLA and PRA bias did not reach significance when the BA analysis was carried out with data in absolute terms (bias of 0.05 g (*p* = 0.73) for PLA, and 8.8 rad/s^2^ (*p* = 0.06) for PRA). The limits of agreement were relatively narrow for both PLA and PRA, which, in combination with the LCCC value of 0.997, showed the mouthguard to strongly agree with the measurements made by the reference sensors. In a practical application, such as field use of the mouthguard, the observed peak acceleration errors of −7 to 6% for PLA and −5 to 7% for PRA are small, and likely to be insignificant compared to other potential sources of error. Greybe et al. carried out a BA analysis, reporting systematic bias of 2.5 g and −0.5 rad/s for PLA and PRV, respectively [52]. Very few other studies regarding sporting head-impact sensors utilised BA analysis, with most using only a linear correlation instead. BA analysis is useful for studies such as these, as it illustrates, in a more easily digestible way, the measurement errors of the device being validated, whilst exposing any under- or overestimation bias.

The furthest outlier in the present PRA BA analysis was due to an underestimation of the PRA by the mouthguard during a rear boss, 40 g, 15 ms impact. Unlike the outlier shown in Figure 4 and Figure 5, the associated RMS error was low, indicating a close match of the shape of the time-series trace between the mouthguard and the reference. The RMS error was useful for investigating the shapes of the two time-series traces. This is important, as many of the metrics for determining the injury potential, such as 15 ms Head Impact Criterion (HIC_15_), Head Impact Telemetry severity profile (HIT_sp_), and other kinematic injury predictors, rely on, and are sensitive to, the shape of the impact-acceleration trace. In previous studies, Camarillo et al. calculated the RMS over 25 data points (24.4 ms) centred about the peak of the impact, assuming this would capture the entire impact [43]. Greybe et al. used similar methodology, with the RMS being taken over the duration of the ‘impact part of the trace’ [52]. The longest impacts reported in this study were 23.5 ± 6.7 ms, with the shortest at 12.1 ± 5.8 ms, which were comparable with those achieved in the present study (9.7–38 ms). The time over which the PLA occurred does not encapsulate the time over which the PRA occurred, nor did it account for the total duration, where a significant amount of excitation existed within the acceleration trace.

For comparison with other studies, ‘Impact’ RMS values were used due to their shorter duration and closer resemblance to durations used in other studies. Camarillo et al. reported average RMS (NRMS) errors of 3.9 ± 2.1 g, (9.9 ± 4.4%) and 202 ± 120 rad/s^2^ (9.7 ± 7%) for linear and rotational acceleration, respectively [43]. Greybe et al. found RMS (NRMS) errors of 4.3 ± 3.5 g (13.1 ± 9.9%) for linear acceleration [52]. Those found for the HitIQ Nexus A9 mouthguard were smaller than those in previous literature, with RMS (NRMS) values of 1.0 ± 0.6 g, (3.0 ± 1.2%) and 47.4 ± 35.0 rad/s^2^, (3.4 ± 1.3%) for linear and rotational acceleration, respectively. When the ‘Full’ time period was used for the RMS error calculation, consistently higher values were achieved, reaching statistical significance for both linear and rotational accelerations. For linear acceleration, this was likely due to the tail of the time-series data trace having a much higher signal-to-noise ratio than that across the impact peak. For rotational acceleration, however, the difference was likely due to the increased amount of large numbers in the time-series data trace across additional peaks on either side of the maximal, rather than excess noise. It should be noted that the effect sizes of the differences in RMS and NRMS errors between the impact and full durations were quite low (0.18–0.56). Similar to the systematic bias displayed in the BA analysis, in a practical setting, these RMS differences would likely be insignificant compared to potential errors from external sources.

The small RMS errors, strong linear correlation, and strong agreement between measurements made by the mouthguard and reference sensors demonstrated the high comparability of peak accelerations and shapes of the acceleration time-series traces between the mouthguard and reference sensors. This study, like those conducted previously, had its limitations. This study was conducted in a controlled laboratory, on a rigid-body headform. This differed from the conditions imposed on the mouthguard during use in sports; for example, through relative motion of the mandible [43], decoupling from the upper jaw due to saliva lubrication, or noise from nonhead impact events. The testing method only incorporated a limited range of PLA values, from 15.2–83.9 g, with a visible break in continuous values from 40–80 g (Figure 3). Rigid coupling between the mouthguard and dentition were assumed during testing and were confirmed by visual inspection after every impact. Future studies should investigate the effects of high- energy, low-duration impacts, as is seen with previous research [43,52,53]. Additionally, a linear impactor could be employed instead of a drop-test rig in order to replicate a greater range of impact locations and scenarios.

## 5. Conclusions

This study revealed that the PLAs, PRAs, and shapes of the time-series acceleration data measured by the HitIQ Nexus 9 instrumented mouthguard closely matched those measured by the reference sensors inside the ATD headform. The methods for impact testing were limited, as only certain impact locations could be tested under certain scenarios with longer impact durations. The results reported in this study only hold up under the assumption that the head is a rigid body. This, however, shows promising results for accurately measuring and investigating head impacts seen in a sporting context, with lower PLA, PRA, and RMS errors than for other instrumented mouthguards previously validated in a laboratory setting.

## Figures and Tables

**Table 1:** Drop heights (m) and foam combination (A–L) (Appendix A and Appendix B) for each impact scenario tested. Those unable to be tested are left blank.

PLA (g)	Duration (ms)	Forehead	Front Boss	Side	Rear	Rear Boss
20 g	15 ms	0.05 (E)	0.11 (A)	0.08 (E)	0.06 (L)	0.08 (I)
	30 ms	0.39 (C)	0.46 (C)	0.60 (C)	0.51 (C)	0.53 (C)
	60 ms	1.16 (H)	1.04 (H)	-	-	-
40 g	15 ms	0.14 (F)	0.24 (B)	0.20 (F)	0.15 (L)	0.17 (F)
	30 ms	0.64 (J)	1.60 (D)	-	-	-
80 g	15 ms	0.65 (G)	-	-	0.63 (K)	-

**Table 2:** Measurement statistics.

Metric	Measurement	Linear	Rotational
Lin’s Concordance correlation coefficient	Peak acceleration	0.997	0.997
LCCC 95% confidence interval	Peak acceleration	(0.996, 0.998)	(0.995, 0.998)
R^2^ value	Peak acceleration	0.996	0.994
Linear regression equation	Peak acceleration	1.04x − 1.28	0.98x + 31.2
Mean (SD)	Impact RMS	0.98 (0.64)	47.41 (34.99)
Mean (SD)	Impact NRMS	3.01 (1.22%)	3.44 (1.33%)
Mean (SD)	Full RMS	1.28 (0.75)	52.20 (31.60)
Mean (SD)	Full NRMS	3.85 (0.92%)	3.85 (0.76%)
Mean (SD) relative peak value error	Peak acceleration	2.56 (2.16%)	2.82 (2.54%)

## Data Availability

Not applicable.

## Appendix A

**Table A1:** Description of foams used for testing.

Foam Name	Thickness (mm)	Manufacturer	Abbreviated Name
Confor Green	13	Trelleborg	CG
Sunmate	12	Dynamic Systems Inc.	SM
LD45	18	Zotefoams	-
LD29	15	Zotefoams	-
HD30	10	Zotefoams	-
EV30	14	Zotefoams	-
LD15	10	Zotefoams	-
LD32	10	Zotefoams	-
Zotefoam Blue	10	Zotefoams	ZFB
Perspex	2	-	P

## Appendix B

**Table A2:** Foam combinations used to achieve PLA and durations for testing.

A	CG
B	P, 3x SM
C	3x SM, CG
D	CG, P, 5x SM, CG, P
E	EV30
F	LD29
G	EV30, ZFB, P, CG
H	6x SM, 3x CG, EV30, ZFB, LD29
I	LD32
J	P, EV30, LD29, ZFB, LD45
K	LD45, P, HD30
L	P, HD30
